# Interfacial characteristics, colloidal properties and storage stability of dairy protein-stabilized emulsion as a function of heating and homogenization

**DOI:** 10.1039/d0ra00677g

**Published:** 2020-03-25

**Authors:** Quanyang Li, Zhengtao Zhao

**Affiliations:** College of Light Industry and Food Engineering, Guangxi University Nanning 530004 China zhengtaozhao85@gmail.com

## Abstract

This research investigated the influence of processing history on physicochemical properties of dairy protein-stabilized emulsions. Emulsions were heated (UHT) either before or after a single homogenization (UHTSH, SHUHT) or homogenized both before and after heating (double homogenization, DHUHT). The results demonstrated that UHT treatment increased the protein load at the oil/water interface while homogenization prior to UHT (SHUHT) inhibited displacement of protein by surfactant molecules, and this emulsion exhibited higher interfacial protein coverage and wider size distribution compared to the emulsion produced by UHTSH. The use of the double homogenization with UHT resulted in emulsion droplets with the smallest average size and lowest concentration of unabsorbed protein. However, no difference in the protein load in a specific area was noticed between emulsions produced by DHUHT and SHUHT. When changes of surface tension at the air/water interface were measured using a drop tensiometer, SHUHT emulsion showed the fastest decrease of surface tension due to the occurrence of a lower level of surfactant displacement where more surfactant was available for fast adsorption. Emulsions prepared with DHUHT or UHTSH decreased the surface tension in a slower speed than SHUHT. During storage, partial coalescence of emulsion droplets was observed for emulsions produced with single homogenization, regardless of whether this was carried out before or after heating. Double homogenization formed more stable emulsions than single homogenization. This work clearly showed that it is possible to tailor physico-chemical functionalities of dairy protein-based emulsions by controlling the interactions between proteins or with surfactants during processing.

## Abstract 2

This research investigated the influence of processing history on physicochemical properties of dairy protein-stabilized emulsions.

## Graphical abstract

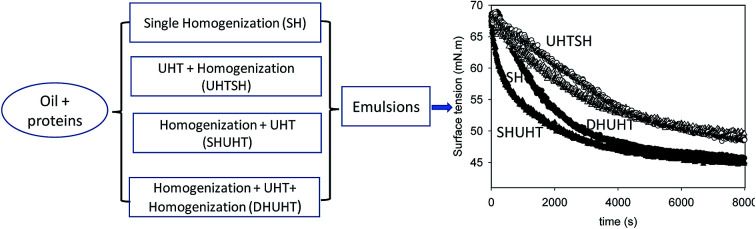

## Introduction

The physico-chemical properties of dairy protein-based emulsions are critical to the stability, functionality and final structure of dairy products. In particular, dairy emulsions are often used to create the structure of aerated dairy products, such as whipped cream and ice cream. Such systems rely heavily on partial coalescence of the fat globules for the development of structure during whipping.^1,2^ Generally, foam formation and foam stability of dairy protein based emulsions depend on many different factors, such as serum viscosity, emulsion droplet size, and the interactions of ingredients (fat, emulsifiers, and stabilizers) in the continuous phase and at the interface.^3^ Coating of the emulsion droplets at the oil/water interface and their adsorption at the air–water interface during whipping also are strongly affected by the composition and processing history of the emulsions.^4^

During the preparation of emulsions, protein molecules adsorb onto the surface of oil droplets, providing the steric and electrostatic repulsion forces necessary to prevent their aggregation.^5^ The structure of protein molecules and their assembly, as well as the overall composition of the interface, determine the thickness of the film and the stability of droplets.^6^ Proteins with compact structures have poorer surface activity and emulsifying capacity than the ones with a more disordered structure. Milk proteins, such as caseins are often used as emulsifiers as they have specific hydrophobic and hydrophilic regions which facilitate protein adsorption at the interface, and allow for areas to protrude in the aqueous phase causing increased electrostatic and steric stabilization.^7,8^

In emulsions containing various emulsifiers, the composition of the interface may vary with time or with processing history, as during homogenization, larger protein complexes may be present in larger amount, but then be displaced over time by other surfactant molecules would achieve a lower interfacial tension.^9^ Previous work has reported the competitive adsorption behaviour of milk proteins (caseins and whey proteins) at the oil/water interface.^10,11^ At temperatures lower than 40 °C, caseins are more readily to adsorb onto the oil/water interface compared to whey proteins, as caseins have a higher proportion of hydrophobic residues and more flexible molecule structures.^12^ The addition of sodium caseinate to a whey protein stabilized emulsion was shown to displace all the adsorbed α-lactalbumin and part of β-lactoglobulin from the interface.^12^ Furthermore, no adsorption of whey proteins was observed when whey protein isolate (WPI) was added to emulsions stabilized by caseinates.^13^

Different adsorption behaviours have been reported with heating treatments, which can cause major changes in protein structure or in the assembly of aggregates. Irreversible denaturation and increased surface hydrophobicity of whey proteins happen when heating is carried out at temperatures higher than 70 °C.^7^ In this case, caseins cannot displace whey proteins on the emulsion surface, and heat denatured whey proteins can displace α_s1_- and β-casein fractions when they were added to a sodium caseinate-stabilized emulsion.^10,11^

Processing history will ultimately affect the composition of the proteins at the emulsion interface. Moreover, small molecular weight surfactants could compete with proteins to adsorb to the emulsion surface, and displace the proteins from the interface.^14^ The extent of displacement is affected by the molecular structure of the proteins. For instance, more proteins could be displaced from the fat globules in homogenized milk by small molecular weight surfactants when heat treatment was performed prior to homogenization than after homogenization.^15,16^ This can affect the functional properties of the ingredients, as displacement of proteins decreases the size of fat globules (by forming a thinner interface) and causes changes in the reactivity of the globules.^17^ It can influence both the acid and rennet gelation properties of milk.^15,18^ All these results indicate that it is important to manipulate the protein composition and the structure of the oil/water interface, as changes can result in different textures or other functionalities.

The objective of this research was to understand the effect of homogenization and heating (UHT) treatments on the physico-chemical properties of a model dairy protein-based oil/water emulsion. The order of processing was studied, to better understand how to modulate the size and processing functionality of a model whipping dairy emulsion. The influence of processing on the protein coverage on the oil/water interface, storage stability and the adsorption behaviour of emulsions at the air/water interface was investigated.

## Methods and materials

### Materials

Palm kernel oil (Aarhus Inc., New Jersey, US) was used as oil phase. Milk protein concentrate (MPC) and sodium caseinate (NaCas) (Alanate 180, Fonterra Co-operative Group Ltd., Auckland, New Zealand) were provided by Gaylea Co-op (Guelph, ON, Canada). Mono- and di-glycerides (Lonza Group, Basel, Switzerland) and sodium stearoyl lactylate (Corbion Food Ingredients, Kansas, US) were used as surfactant. High fructose corn syrup (Gaylea, Guelph, ON, Canada) was used as bulking agent.

### Preparation of emulsions

A 20% (w/w) oil in water emulsion was prepared by adding palm oil to the aqueous phase which contains 53.2% distilled water, 0.5% (w/w) MPC, 0.5% (w/w) NaCas, 0.25% (w/w) mono- and di-glycerides, 0.5% (w/w) sodium stearoyl lactylate and 0.05% (w/w) κ-carrageenan. The mixtures were agitated at 55 °C for 1 h to ensure full dissolution and hydration of protein molecules. The high fructose corn syrup (25%, w/w) was then added and agitated for another 10 min to ensure thoroughly mixing.

The emulsions were homogenized and heated in a laboratory-scale MicroThermics processing system (UHT/HTST Lab-25 EDH, MicroThermics Inc., Raleigh, NC, USA) and a NS2006H homogenizer (GEA Niro Soavi, Parma, Italy) were used to prepare the emulsions under different conditions, which were summarized below:

Emulsion 1 (SH): single homogenization (2000/500 psi, 72 °C)

Emulsion 2 (UHTSH): UHT (123 °C, 16 s) + single homogenization (2000/500 psi, 72 °C)

Emulsion 3 (SHUHT): single homogenization (2000/500 psi, 72 °C) + UHT (123 °C, 16 s)

Emulsion 4 (DHUHT): single homogenization (2000/500 psi, 72 °C) + UHT (123 °C, 16 s) + single homogenization (2000/500 psi, 72 °C)

After preparation, all emulsions were cooled using running water and 0.02% (w/w) sodium azide was added immediately to prevent the growth of bacterials.

### Size distribution

Mastersizer 2000S (Malvern Instruments Inc., Southborough, MA) was used to determine the droplet size distribution of emulsions. This laser diffraction analyser using the Mie theory to calculate the size distribution with the assumption of particle sphericity. In this research, the refractive indices used for palm kernel oil and water were 1.455 and 1.330 respectively and the reported data was volume frequency based distribution. The surface-weighted mean diameter [*D*_[3,2]_] was used to interpret the particle size data. Each sample was measured in triplicate. The measurements were performed at 25 °C.

### Zeta potential

All the emulsions were diluted 1000× with filtered (0.22 μm) Milli-Q water and then determined by laser Doppler electrophoresis using the Nano-S Zetasizer (Malvern Instruments). Each measurement was obtained from the average of 20 readings and the results were expressed in absolute values (mV).

### Rheological properties

The rheological properties of the emulsions were determined using a controlled stress rheometer (Paar Physica MC 301, Anton Paar, Graz, Austria) equipped with a Peltier temperature controller and a concentric cylinder geometry. Samples (20 mL) were pipetted to the cylinder, allowed to equilibrate for 2 min, and subjected to a steady flow test (shear rate ramp from 10–300 s^−1^). All tests were conducted at 25 °C.

### Protein load

To determine the amount of protein adsorbed to the surface of oil droplets, the cream phase and aqueous phase were separated by centrifugation at 10 000 g for 30 min (Eppendorf centrifuge 5415D). The aqueous phase was then removed using a syringe and the weight of the cream phase was weighed. The total protein concentration of emulsions and protein content of the cream phase were determined using a Dumas nitrogen analyzer (FP-528, Leco Inc. Lakeview Avenue, St. Joseph, MI) and protein content was calculated using 6.38 as conversion factor. The percentage of the protein loaded to the interface of the droplet was calculated using the following equation.

1 *E* (%) = (*C*_cream_ × *D*)/*C*_emulsion_

where *E* is protein load, *C*_cream_ and *C*_emulsion_ are the protein concentration in the cream phase and the emulsion respectively. *D* is the weight based percentage of cream phase to the emulsion, which was calculated using eqn (2).

2 *D* = (*M*_1_ − *M*_e_)/(*M*_2_ − *M*_e_)

where *M*_e_ is the weight of empty Eppendorf tube. *M*_1_ is the weight of cream phase plus Eppendorf tube. *M*_2_ is the weight of emulsion plus Eppendorf tube.

### Drop shape tensiometry

To investigate the adsorption behavior of droplets produced with different processing at the air/water interface, the changes of the interfacial tension (*γ*) and elastic modulus (*E*) were measured using drop shape tensiometry (Tracker, IT Concept, Longessaigne, France) at room temperature. The emulsions were diluted 2000 times with Milli-Q water and mixed well in an optical glass cuvette. A 5 μL air bubble was then formed at the tip of the J-shaped syringe needle immersed in the cuvette. Drop volume was consistently controlled using a volume control regulation program. The changes of interfacial tension (*γ*) was calculated by analyzing profiles every 0.5 s according to the Laplace equation by the software.^19^ The dilatational viscoelasticity measurements were conducted after an equilibration period of 3 h. After that, a sinusoidal oscillatory changes of volume/surface area of the air were performed with the strain amplitude kept constant at 10% (Δ*A*/*A* = 0.1, where *A* is the droplet surface area). The surface dilatational modulus was calculated from the change in interfacial tension (d*γ*) relative to the change of air surface area (d*A*), as described in eqn (3).^19^

3 *E* = d*γ*/(dln *A*)

### Storage stability

The emulsions were stored in the fridge (4 °C). The changes of rheological properties and the size distribution at different storage time (0, 5, 10, 20 days) were used to assess the stability. All samples were equilibrated for at least 2 h at room temperature before measurement.

## Results and discussion

### Effect of processing history on the size distribution of emulsions

**Fig. 1:**
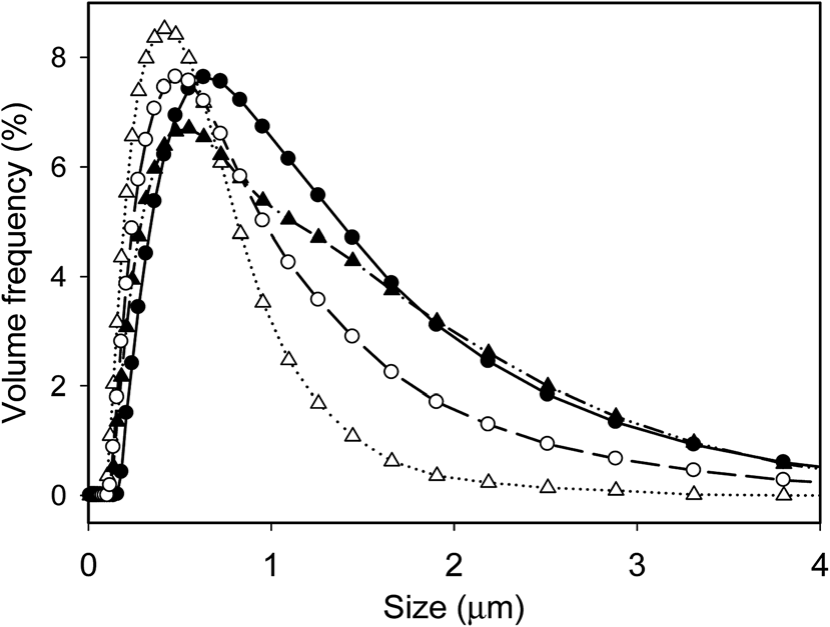
Particle size distribution of emulsions prepared by different processing histories: SH (filled circles), UHTSH (empty circles), SHUHT (filled triangles) and DHUHT (empty triangles).

All emulsions had a similar pH value of around 6.40 (Table 1), indicating the pH of emulsion was not influenced by the processing history. Similarly, all emulsions had a zeta potential value of around −43 mV without any significant difference. On the other hand, emulsions produced by different processing history exhibited noticeable different size distribution, as indicated in Fig. 1. The control emulsion (SH) had the widest size distribution and the highest *D*_[3,2]_ value of 571.7 ± 3.5 nm. UHT treatment shifted the peak to smaller size direction due to exposure of buried hydrophobic residues after heat treatment, particularly for whey proteins which were globular and susceptible to heating although the amount of whey proteins (0.1%) was much lower than caseins (0.9%).^7,20^ Moreover, although the structure of caseins are more flexible and remarkably heat-stable due to the lack of tertiary structure,^21^ it was also reported that heat treatment could lead to irreversible changes to the structure of caseins and those changes contribute to the improved emulsifying capacity,^22,23^ which was in consistent with our results, where the emulsion prepared with UHT treated proteins had smaller size and higher specific surface area than the emulsion prepared with unheated proteins.

**Table 1:** pH, *D*_[3,2]_, specific surface area and zeta potential of emulsions prepared with different processing histories. Values are means from triplicate experiments, ±standard deviation. Average values in the same column with a different superscript letter are significantly different (*p* < 0.05)

	pH	*D*_[3,2]_ (nm)	Specific surface area (m^2^ g^−1^)	Zeta potential (mV)
SH	6.50 ± 0.09^a^	571.7 ± 3.5^a^	10.5 ± 0.0^a^	−42.7 ± 3.9^ab^
UHTSH	6.44 ± 0.05^a^	425.0 ± 20^b^	14.7 ± 0.1^b^	−42.6 ± 1.1^ab^
SHUHT	6.40 ± 0.01^a^	471.0 ± 1.0^c^	12.7 ± 0.0^c^	−43.0 ± 0.2^b^
DHUHT	6.40 ± 0.08^a^	341 ± 28^d^	17.3 ± 0.4^d^	−45.6 ± 0.3^a^

Unlike previous research, where it was illustrated that the order of processing (H-before, H-after) did not affect the apparent size of fat globules,^13^ the results from this research showed that emulsion produced with heating prior to homogenization exhibited a smaller size compared to heating after homogenization (Fig. 1). The different sizes were related to the interfacial composition as thicker protein layer on fat globule membrane has been observed previously when heating was performed after homogenization than before homogenization.^16^ Moreover, the presence of small surfactant molecules could compete with protein molecules at the surface of oil droplet,^24^ resulting in further changes of interfacial membrane composition. The significant smaller size for emulsion prepared with UHTSH suggested that a higher extent of protein was replaced by surfactants compared to the emulsion prepared with SHUHT. Double homogenization further broke emulsions into smaller sizes and the produced emulsion had the smallest *D*_[3,2]_ value of 341 ± 28 nm and the highest specific surface area.

### Effect of processing history on the protein load on the surface of oil droplet

**Fig. 2:**
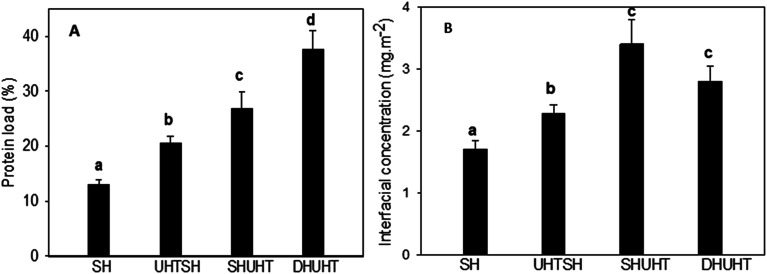
Percentage of protein loaded to the surface of oil droplet (A) and interfacial protein concentration of emulsions prepared with different processing histories (B).

To better characterize the effect of processing history on the interfacial properties of emulsion droplets, the amount of protein loaded on the oil/water interface was determined, as shown in Fig. 2. Only about 13% of protein was found to be present on the oil/water interface for the SH emulsion, which was the lowest among all emulsions (2A). UHT treatment increased the loading of protein molecules, which was due to the exposure of hydrophobic groups and formation of new peptides after UHT treatment.^25^ Compared to the emulsion produced with UHTSH, more protein molecules were present in the oil phase for the emulsion prepared with SHUHT. The results were in correspondence with previous publications.^10,15^ UHT treatment prior to homogenization enhanced the formation of protein aggregates through hydrophobic interaction and formation of disulphide bonds,^26,27^ which decreased their adsorption speed on the increased interface during homogenization when competing with small surfactant molecules. On the contrary, when UHT was performed after homogenization, more proteins were adsorbed to the increased oil/water interface during homogenization, and subsequent UHT treatment facilitated intermolecular interaction of protein molecules at the oil/water interface. Moreover, the capacity of surfactant to replace protein molecules was decreased when UHT was performed after homogenization.^15^ In general, it can be concluded that the spreading and rearrangement of adsorbed protein molecules were different between UHTSH and SHUHT emulsions.^28^

The interfacial protein concentration (mg protein per m^2^ surface area) was calculated by dividing the specific surface area of the oil droplet determined by the mastersizer and the results are shown in Fig. 2B. The protein coverage for the SH emulsion was around 1.7 mg m^−2^. After UHT treatment, the interfacial concentration of proteins increased to 2.3 and 3.4 mg m^−2^ for emulsions produced using UHTSH and SHUHT respectively. The results further confirmed the hypothesis that heat treatment inhibited the capacity of surfactant to replace protein molecules at the surface of oil droplet. The inhibition effect was stronger when the heat treatment was performed before homogenization. Although much less proteins were present on the surface of droplet when UHT was performed before homogenization, UHTSH emulsion exhibited a narrower size distribution and a smaller average size compared to SHUHT emulsion. In contrast, double homogenization significantly decreased the average size of oil droplet (Table 1). However, no significant difference in the interfacial protein concentration was observed compared to that of SHUHT.

### Rheological properties

**Fig. 3:**
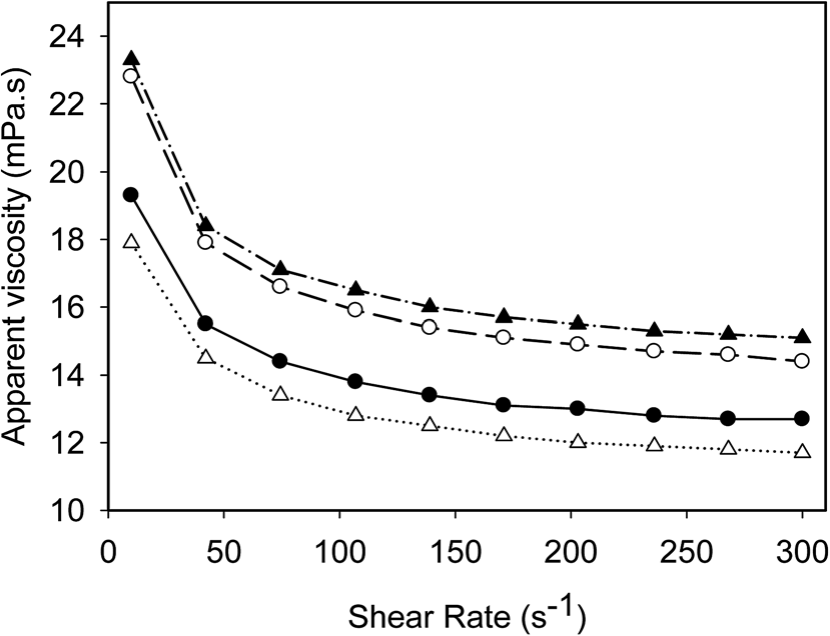
Rheological properties of emulsions prepared by different processing histories: SH (filled circles), UHTSH (empty circles), SHUHT (filled triangles) and DHUHT (empty triangles).

Flow behaviours of emulsions prepared at different conditions are summarized in Fig. 3. All emulsions exhibited similar flow behaviour. Emulsions produced with UHTSH and SHUHT had highest viscosities, followed by SH emulsion and DHUHT emulsion. The higher viscosity values for UHTSH and SHUHT samples were probably due to heat-induced structural changes and reorganization of protein molecules at the oil/water interface, which exposed more exposed hydrophobic residues and increased the probability of aggregation.^29^ The lowest viscosity was observed for the emulsion produced with DHUHT, owing to the smallest size distribution produced by double homogenization technique (Table 1). In all cases, the apparent viscosities reduced dramatically at low shear rate (<150 s^−1^) and reached to a stable value at higher shear rate (150–300 s^−1^). It was reported that this shear-thinning behaviour of concentrated oil/water emulsion was a result of the formation of aggregates or clusters.^30^ The stable viscosity values at high shear rate (>150 s^−1^) indicated that the interparticle weak forces could be disrupted. The results were in fully agreement with previous research performed on the dairy-protein based emulsion products.^2,31^

### Adsorption behaviour of emulsions onto the air/water interface

**Fig. 4:**
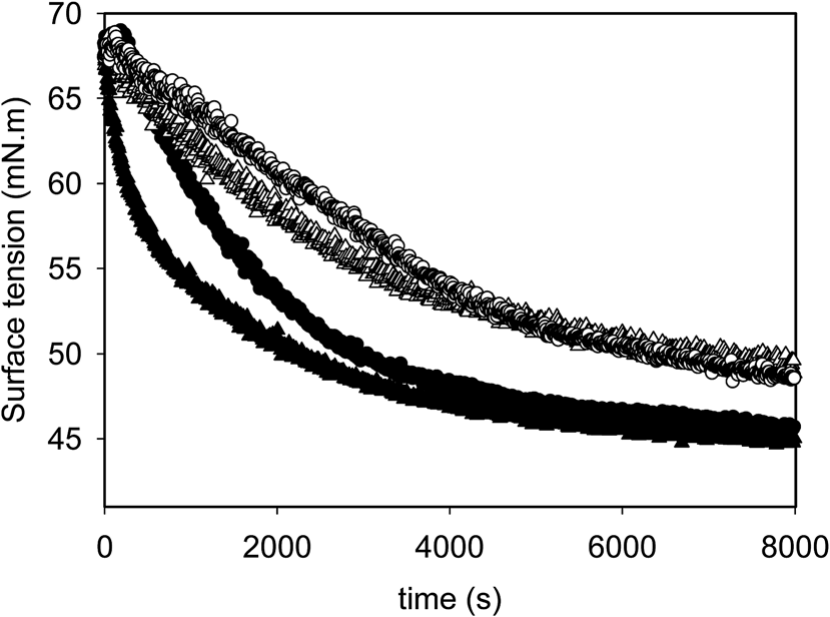
Changes of surface tension of the air/water interface as a function of time for emulsions prepared with different processing histories: SH (filled circles), UHTSH (empty circles), SHUHT (filled triangles) and DHUHT (empty triangles).

Fig. 4 summarizes the adsorption behaviour of different emulsions at the air/water interface as a function of time. The emulsions were diluted 2500 times using Milli-Q water and the dynamic surface tension (*γ*) of the air bubble was monitored with time. In all cases, the *γ* decreased with time, indicating the adsorption of droplet on the air/water interface. The emulsions produced from different processing histories showed different adsorption manners. The value of *γ* at plateau was also calculated by plotting values of *γ* as a function of 1/sqrt(*t*), as summarized in Table 2. No significant difference in the final surface tension was observed for all emulsions, indicating the final surface tension was dominated by the spreading of oil droplet instead of surfactants or protein molecules, which has been reported to involve in the rupture of protein surface layer after the emulsion droplets were brought to the air/water interface.^32^

**Table 2:** Interfacial tension and elasticity modulus of different emulsions prepared with different processing histories after equilibrium reached. Values are means from triplicate experiments, ±standard deviation. Average values in the same column with a different superscript letter are significantly different (*p* < 0.05)

	Surface tension (mN m)	Elasticity modulus (mN m)
SH	38.8 ± 2.7^a^	27.1 ± 2.8^a^
UHTSH	37.4 ± 2.0^a^	28.7 ± 1.0^a^
SHUHT	35.6 ± 5.2^a^	31.3 ± 3.0^a^
DHUHT	41.7 ± 1.9^a^	42.6 ± 2.5^b^

From the initial slopes of the adsorption curves, which is an indication of the adsorption rate, the adsorption of different emulsions follows the sequence of SHUHT > SH > UHTSH = DHUHT. There were three possible components in the emulsions could contribute to the decrease of the *γ* with time: surfactants, proteins and the oil droplets. The rapid decrease of the *γ* for SHUHT was due to the presence of high amount of free surfactant molecules which can adsorb onto the air/water interface at a much faster speed. In the case of SH, where no heating was performed, protein molecules at the surface of emulsion droplet could be replaced by surfactants and was released to the serum phase. The presence of high amount of aqueous protein molecules contributed to the fast decrease of *γ*. Moreover, when unheated emulsion was introduced to the air/water interface, protein layers around emulsion droplet were not adequately coherent to resist the forces that needed for the spreading,^32^ resulting in the fast reduction of *γ*. When UHT was performed prior to homogenization, a more coherent protein layer was formed at the surface of oil droplets, inhibiting the adsorption and spread of emulsion droplets at the air/water interface. Double homogenization before and after UHT treatment produced droplets with smaller size and higher interfacial protein coverage (Fig. 2). Less surfactants and protein molecules were present in the aqueous phase as most of them were transferred to the increased area of oil/water interface during second homogenization process. In addition, the produced DHUHT emulsion also had more coherent protein coverage. The combination effects of low concentration of free surfactants and proteins as well as more coherent interfacial coverage resulted in the slow decrease of *γ* at the air/water interface. The results clearly demonstrated that the processing history had a significant influence on the distribution of protein and surfactant molecules between the oil and aqueous phases.

Moreover, the interfacial elasticity was measured after 16 000 s of equilibration when a sinusoidal compression was applied to the air bubbles. The amplitude used was within the linear viscoelastic range. The results of surface elastic modulus of the air bubbles, which represents the resilience of adsorbed film and is correlated to the foam stability, was also calculated and presented in Table 2.^33,34^ The control SH emulsion showed an elastic modulus value of 27.1 ± 2.8 mN m and no significant differences were detected between UHTSH and SHUHT emulsions, which was probably due to the produced droplets have large size thus reduced the amount of emulsion droplets adsorbed on the air/water interface. It was also reported the presence of protein molecules at the air/water interface inhibited the adsorption and spreading of emulsion droplet.^32^ On the other hand, a significant higher elasticity value of 42.6 ± 2.5 mN m was observed for the DHUHT emulsion, indicating more droplets were adsorbed on the interface. Apparently, the emulsion produced with DHUHT treatment had a better capacity in maintaining the foam stability, although it adsorbed onto the air/water interface in a slower manner. In summary, the adsorption behaviour of emulsion at the air/water surface and the elastic modulus of the interface can be modified by processing.

### Storage stability

**Fig. 5:**
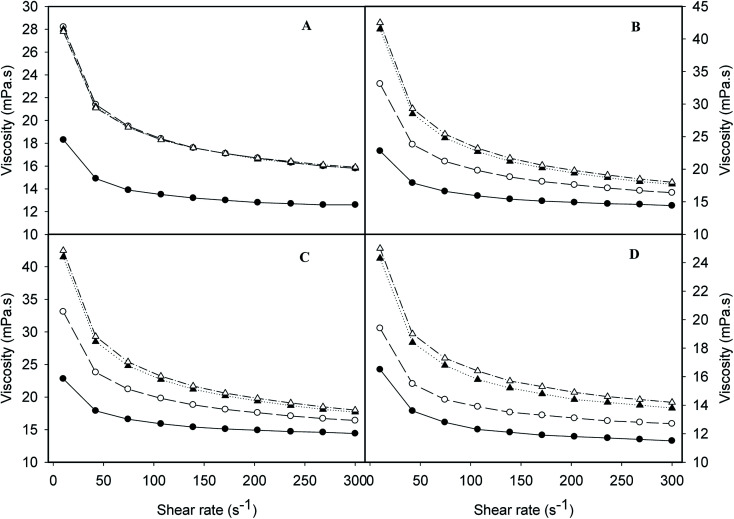
Changes of viscosity at different storage time (4 °C): 0 day (filled circles), 5 days (empty circles), 10 days (filled triangles) and 20 days (empty triangles) for emulsions prepared with different processing histories: SH (A), UHTSH (B), SHUHT (C) and DHUHT (D).

**Fig. 6:**
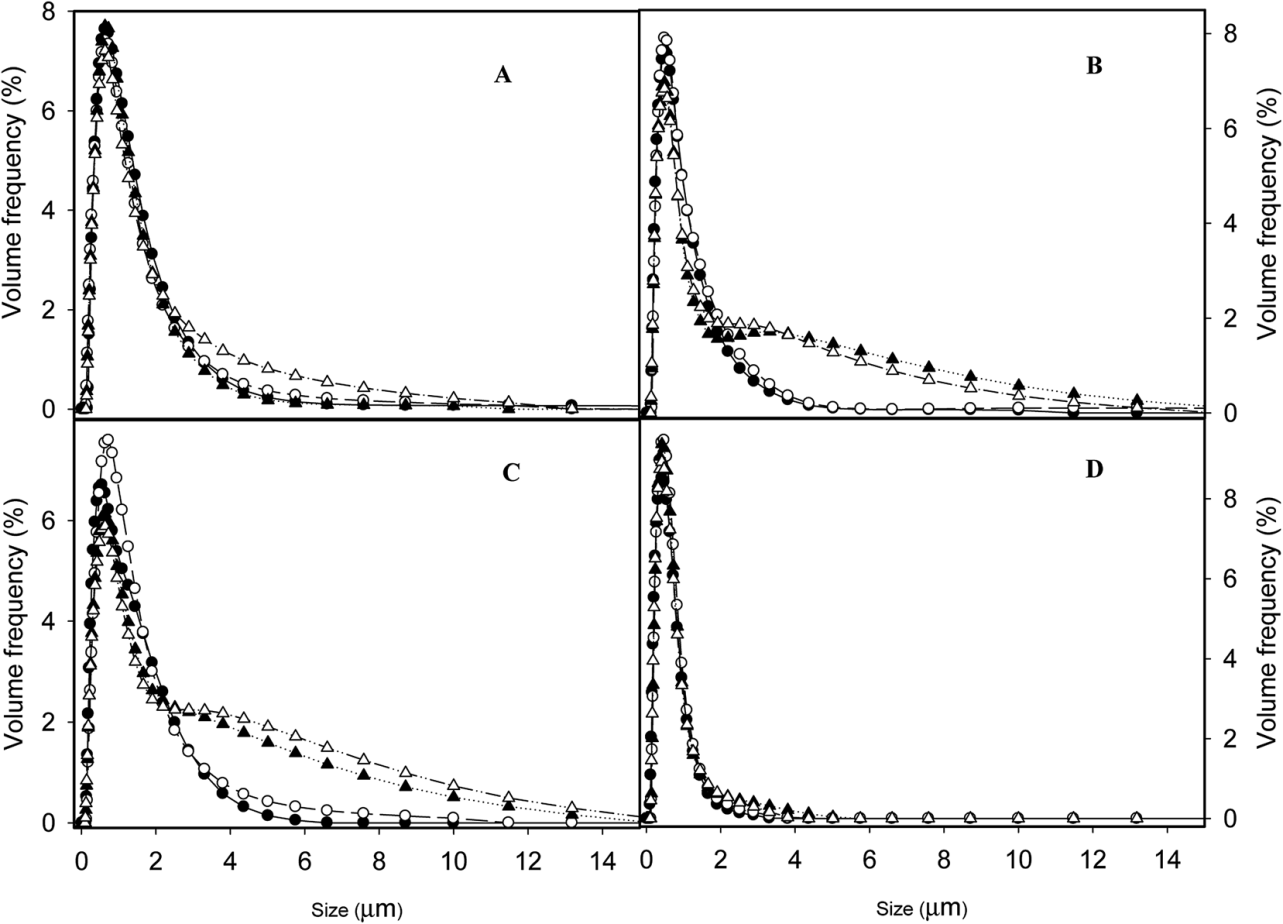
Changes of size distribution at different storage time (4 °C): 0 day (filled circles), 5 days (empty circles), 10 days (filled triangles) and 20 days (empty triangles) for emulsions prepared with different processing histories: SH (A), UHTSH (B), SHUHT (C) and DHUHT (D).

The changes of rheological properties and size distribution during storage (4 °C) were also measured, as summarized in Fig. 5 and 6. A rapid increase of the viscosity was observed at 5 days' storage for SH emulsion, probably due to the low coverage of protein molecules at the oil/water interface, which provided lower repulsions compared to other emulsions. The viscosity kept stable up to 20 days (Fig. 5A). On the other hand, for the UHTSH, SHUHT and DHUHT emulsions, the viscosities increased up to 10 days (Fig. 6B–D). The increase of viscosity during storage had been ascribed to the aggregation or the coalescence of droplets due to increased exposure of hydrophobic residues of protein molecules derived from UHT treatment, which increased the interaction between emulsion droplets.^31,35^ During storage, the emulsion droplet slowly approached and connected with each other through hydrophobic interactions. The plateau viscosity suggested that no further aggregation occurred after 10 days, storage.

Fig. 6 summarizes the changes of the size distribution of different emulsions during storage. The emulsion produced with SH showed notable storage stability and no change of the size was detected up to 20 days (Fig. 6A), which was in correspondence with the result of viscosity (Fig. 5A). The increased viscosity after 5 days was a result of aggregation of droplets, which can be disrupted when diluted in water under agitating. No irreversible coalescence occurred during a storage time of 20 days. For the UHTSH and SHUHT emulsions, the size distributions were stable at day 5. However, the occurrence of big droplets was detected (Fig. 6B and C) at day 10, which was irreversible during agitation process, indicating the occurrence of partial coalescence. No further increase of droplets size was observed at 20 days. Apparently, UHT treatment changed the protein arrangement on the surface of droplet resulting in the increased interaction between emulsion droplets. For those two emulsion samples, the increase of the viscosity at day 5 was a result of the aggregation of droplets while the increase from day 5 to day 10 was mainly from the partial coalescence. Double homogenization significantly improved the emulsion stability compared to UHTSH and SHUHT. No change of size distribution was detected during the storage up to 20 days. The increased stability was a result of more homogeneous size distribution and more coherent interface coverage (Fig. 1 and 2), which inhibited the coalescence of emulsion droplets. In this case, the increase of viscosity during storage (Fig. 5D) was solely from the aggregation of droplet. Overall, the changes of size distribution were in fully agreement with the development of viscosity during the storage. Processing history significantly changed the structure and arrangement of protein molecules on the surface of oil/water interface, which further changed the colloidal properties and functionalities of emulsion droplet.

## Conclusion

The structure and distribution of protein and surfactant molecules between the aqueous phase and the oil phase play an important role in the stability and foamability of dairy protein-stabilized emulsions. The results from this research illustrated that the physico-chemical properties of emulsion are influenced by the processing history. More proteins were transported to the surface of oil droplet when heating was performed. The ability of surfactants molecules to replace the protein molecules was decreased when the UHT treatment was performed after homogenization compared to before. The changes of distribution of protein and surfactants molecules between aqueous phase and oil phase significantly influence the adsorption behaviours of emulsions onto the air/water interface. SH and SHUHT emulsions showed higher ability in decreasing the surface tension, while DHUHT and UHTSH emulsions decreased the surface tension at a much slower speed. The combination of single homogenization and UHT treatment decreased the stability of emulsions, where partial coalescence was detected after storage of 10 days. On the other hand, SH and DHUHT emulsions showed notable stability during the storage. The results from this research could provide theoretical guidance for the optimization the production of dairy-protein stabilised products, particularly for the whipped cream products.

## Conflicts of interest

There are no conflicts of interest to declare.

## Supplementary Material
